# Aspirin and lung cancer risk in a cohort study of women: dosage, duration and latency

**DOI:** 10.1038/sj.bjc.6603996

**Published:** 2007-09-25

**Authors:** D Feskanich, C Bain, A T Chan, N Pandeya, F E Speizer, G A Colditz

**Affiliations:** 1 Channing Laboratory, Department of Medicine, Brigham and Women's Hospital and Harvard Medical School, Boston, MA, USA; 2 School of Population Health, University of Queensland, Brisbane, Australia; 3 Gastrointestinal Unit, Massachusetts General Hospital and Harvard Medical School, Boston, MA, USA; 4 Population Studies and Genetic Research, Queensland Institute of Medical Research, Queensland, Australia; 5 Department of Environmental Health, Harvard School of Public Health, Boston, MA, USA; 6 Department of Epidemiology, Harvard School of Public Health, Boston, MA, USA; 7 The Harvard Center for Cancer Prevention, Harvard School of Public Health, Boston, MA, USA

**Keywords:** lung cancer, aspirin, cohort, women

## Abstract

Aspirin may reduce the risk of cancer at some sites but its effect at the lung is unclear. We prospectively examined associations between aspirin use and risk of lung cancer in 109 348 women in the Nurses' Health study from 1980 to 2004. During this time, 1360 lung cancers were documented in participants 36–82 years of age. Aspirin use and smoking were assessed every 2 years. Risk of lung cancer was a non-significant 16% lower for regular aspirin users of one or two tablets per week and a significant 55% higher for users of 15 or more tablets per week compared with women who never regularly used aspirin. Results were similar when limited to never smokers. For both the low and high quantity aspirin users, risk of lung cancer did not decline or increase with longer durations of use, and associations attenuated as the latency period between aspirin assessment and lung cancer diagnosis was lengthened. Our findings, together with those from previous clinical trials and prospective studies, do not provide consistent evidence that aspirin influences the development of lung cancer and further investigation is required with adjustment for smoking.

Regular use of aspirin reduces the risk of colorectal adenoma and cancer (Benamouzig *et al*, 2005), most likely through the inhibition of cyclooxygenase enzymes, restoration of normal apoptosis, and reduction of angiogenesis (Zha *et al*, 2004). However, the influence of aspirin at other tumour sites is less clear. For lung cancer, several meta-analyses with different coverage of published results have offered varying results and interpretations. Of the two that focused solely on lung cancer, aspirin users were found to have a significant 27% lower risk in one (Khuder *et al*, 2005) and a non-significant 9% lower risk in the other (Hernandez-Diaz and Rodriguez, 2007). In two other meta-analyses in which lung cancer was embedded in a wider review of many cancers, the first reported a 16% lower risk for aspirin users that was compatible with no effect or a slightly reduced risk (Gonzalez-Perez *et al*, 2003), and the other, limited to cohort studies, reported no association (Bosetti *et al*, 2006). In general, these meta-analyses concluded that a chemopreventive value of aspirin for lung cancer should be interpreted with caution owing to the limitations of the available studies and the heterogeneity of study designs and results. The authors called for larger studies with better exposure characterisation of dose–response measures and detailed adjustment for smoking. In an attempt to resolve these uncertainties, we examined relations between regular aspirin use and risk of invasive lung cancer among women in the Nurses' Health Study (NHS) cohort, taking into consideration quantity, frequency, and duration of use and latency between exposure and diagnosis, while controlling for detailed smoking characteristics. We placed our results within a wider context by conducting a literature review focused on clinical trials and prospective studies that, like our study, had a quantitative measure of aspirin use and controlled for smoking in analyses.

## METHODS

### Study population

The NHS was established in 1976 when 121 700 female registered nurses, 30–55 years of age, returned a mailed questionnaire. The NHS was approved by the Institutional Review Board of the Brigham and Women's Hospital in Boston. Using mailed questionnaires, participants provided a disease history and information about their personal characteristics and behaviours at baseline, and every 2 years they have updated and extended these data and reported newly diagnosed diseases on follow-up questionnaires. Deaths are commonly reported by families or the postal service and are confirmed through the National Death Index.

For this investigation, analysis began in 1980 when aspirin use was first ascertained, and follow-up for incident lung cancer continued until June 2004. The baseline study population consisted of the women who had not reported a diagnosis of cancer (except non-melanoma skin cancer) and provided information on their smoking status and aspirin use. A total of 109 348 women, 34–82 years of age over the follow-up period, contributed approximately 1.9 million person-years. The follow-up rate for this study population was 95%.

### Lung cancer cases

When lung cancer was reported by a cohort member or identified from a death report, we sought medical records for confirmation and date of diagnosis. Between 1980 and 2004, 1446 incident lung cancers were reported among the women in this study population, of which 1360 were confirmed as cases, with a median age at diagnosis of 65 years (range 36–82 years). The majority of lung cancers were adenocarcinomas (46%), small cell (17%), or squamous cell (15%) tumours. The 86 unconfirmed lung cancers were either metastatic from another site or lacked a follow-up response from the participant and were censored at time of self-report.

### Aspirin assessment

Aspirin use was first assessed in 1980, when participants were asked if they used aspirin in most weeks, and if so, to write the number of years of use and the number of aspirin tablets consumed per week. On subsequent biennial questionnaires, participants were asked if they were a regular aspirin user over the past 2 years and tablets per week and/or days per week of use were assessed with categorical responses. In 2000 and 2002, participants were additionally asked whether they took standard dose (325 mg or more) or low-dose (100 mg or less) aspirin. We converted reported tablets per week of low-dose aspirin into the equivalent tablets of standard dose.

In this analysis, we reclassified participants in each 2-year follow-up cycle by status and quantity (1–2, 3–5, 6–14, ⩾15 tablets per week) of aspirin use. A participant was classified as a current aspirin user if she reported at least 1 tablet per week or 1 day per week of regular use for the last 2 years; a past user if she did not qualify as a current user but had been previously classified as such; or a never user if she had never been classified as a current user during cohort follow-up. Duration was calculated for current users in each follow-up cycle as years of continuous aspirin use.

To determine reasons for aspirin use in this cohort, we had queried a sample of 100 women who reported taking 1–6 tablets per week in 1980, 1982, and 1984 (90% response) and 100 women who reported taking 7 or more tablets per week in those years (92% response). In both groups, the major reasons for aspirin use were headache, arthritis, and/or musculoskeletal pain. Less than 10% of both the lower and higher quantity users cited cardiovascular disease prevention.

### Smoking and other covariates

On the initial NHS questionnaire, participants reported whether they were a current smoker or had ever smoked in the past, the age at which they began to smoke, the number of cigarettes typically smoked in a day, and the age at which they stopped smoking. On each subsequent biennial questionnaire, participants again reported whether they currently smoked and their daily quantity of cigarette use. In this analysis, we reclassified participants in each 2-year follow-up cycle by smoking status, cigarettes smoked per day among current smokers, time since quitting among past smokers, and age at start of smoking among ever smokers. Pack-years were also calculated in each cycle as the product of years of smoking and packs of cigarettes smoked per day.

Height was reported in 1976 and body mass index (BMI in kg m^−2^) was calculated with each biennial report of body weight. Menopausal status and use of oestrogen replacement therapy were updated every 2 years. The total number of hours per week of physical activity was ascertained in 1980 and on eight subsequent questionnaires. Diet was assessed with a food frequency questionnaire six times beginning in 1980 and daily intakes of fruits, vegetables, *α*-carotene, lycopene, vitamin C, vitamin E, total fat, and alcohol were calculated.

### Statistical analysis

Participants contributed person-time from the return date of their 1980 questionnaire until a report of any other cancer except non-melanoma skin cancer, death, or end of follow-up on 1 June 2004. The median follow-up time per participant was 23.7 years. The most recent data on aspirin use and all covariates were used to allocate person-time to the appropriate category for each variable at the beginning of every 2-year follow-up cycle. Participants did not contribute person-time in any follow-up cycle in which they were missing aspirin or smoking status.

Age-adjusted incidence rates of lung cancer were determined within categories of aspirin use and relative risks (RRs) were calculated as the ratio of the rate in each category of use compared with the rate in never users. We used Cox proportional hazards models to adjust simultaneously for age, smoking characteristics, and the other potential confounders. To assess dose–response effects for duration of aspirin use, *P*-values for linear trend were calculated using medians per category.

### Literature review

Relevant papers were sought electronically and by hand. The PubMed database was searched without limits for ‘aspirin and lung and cancer’. Reference lists of all articles of interest were scanned for additional manuscripts and all papers citing two of the earliest relevant publications (Peto *et al*, 1988; Thun *et al*, 1993) were tracked through Science Citation Index. We reviewed all papers with original data that assessed the relation between aspirin use and lung cancer incidence or mortality. We excluded retrospective analyses (Rosenberg 1995; Harris *et al*, 2002; Moysich *et al*, 2002; Muscat *et al*, 2003) because of potential biases in aspirin recall and selection of controls and the observed heterogeneity of results from case-control and cohort studies in previous reviews. We also excluded prospective studies that did not account for smoking (Paganini-Hill *et al*, 1989; Friis *et al*, 2003; Sorensen *et al*, 2003) or had no quantitative measure of aspirin use (Shreinemachers and Everson, 1994; Ratnasinghe *et al*, 2004) because these factors likely influence the association between aspirin and lung cancer. After exclusions, we identified nine studies that met our criteria for inclusion in the review (see Table 4). Confidence intervals (CIs) were missing from one paper (Peto *et al*, 1988) for which we calculated approximate intervals around the RR.

## RESULTS

The characteristics of the women in the study population are shown in Table 1 by status and quantity of regular aspirin use over the 1980–2004 follow-up period. Current and past aspirin users were older than those who never used aspirin on a regular basis (57 and 60 *vs* 53 years, respectively). After adjusting for age differences, the percent of women who smoked varied little between the never (21%) and current (18%) aspirin users, though among the current users, the likelihood of smoking and daily cigarette use increased with increasing aspirin tablets per week. The highest quantity aspirin users had the highest BMI and the post-menopausal women who never used aspirin regularly were least likely to use oestrogen replacement therapy. No differences by aspirin use were detected for the dietary variables nor for the single assessment of exposure to second-hand smoke in 1982 (ie number of adult years spent living with someone who smoked regularly, and hours per week exposed to cigarette smoke from people at home or work) (data not shown).

In the age-adjusted analysis, current aspirin users had a modestly lower risk of lung cancer when compared with women who never used aspirin regularly, and the RR was attenuated and became null after the smoking factors were added to the model (Table 2). However, when we examined the influence of aspirin according to quantity consumed, we observed a non-significant 16% lower risk for current users of 1 or 2 tablets per week (low quantity), a significant 55% increased risk for current users of 15 or more tablets per week (high quantity), and no risk or benefit for women using between 3 and 14 tablets per week. Results were essentially unchanged when all covariates were added to the model or when pack-years were used to control for smoking instead of age at start, years since quit, and cigarettes per day (data not shown). Frequency of aspirin use exhibited an association with lung cancer that was similar to that seen for quantity, with a lower risk for use on 1 day per week (RR=0.85, 95% CI 0.69–1.04) and an increased risk for use on 6 or 7 days per week (RR=1.20, 95% CI 1.00–1.40).

Associations between aspirin use and lung cancer did not differ by smoking status. Even among the never smokers with 128 lung cancers, the association was positive for high quantity users (RR=1.70, 95% CI 0.67–4.31) and inverse for low quantity aspirin users (RR=0.71, 95% CI 0.39–1.30). However, the inverse association was only evident for adenocarcinoma tumours (RR=0.83, 95% CI 0.62–1.10) and in women less than 70 years of age (RR=0.77, 95% CI 0.62–0.95).

Duration of aspirin use was unrelated to lung cancer risk (Table 3). With longer durations, risk neither notably decreased for low quantity users nor increased for high quantity users. We also examined timing of exposure to determine whether earlier use was more relevant than current use for lung cancer aetiology. However, for both low and high quantity users, the strongest associations were those observed for current users (diagnosis within the 2 years between aspirin assessments) and attenuated with increasing latency between aspirin assessment and lung cancer diagnosis.

The clinical trials and prospective studies of aspirin and lung cancer that were included in our review are described in Table 4. Despite the focus on research that included a quantitative measure of aspirin use and accounted for smoking in analyses, results remain inconsistent. The lowest RRs were reported from two studies (Peto *et al*, 1988; Akhmedkhanov *et al*, 2002) that had few lung cancer cases and very imprecise results. The only significant inverse association was the 27% lower risk in women in the Cancer Prevention Study II cohort who used aspirin 1–15 times per month for at least 1 year (Thun *et al*, 1993). Five other studies reported non-significant risk reductions. In the Women's Health Study clinical trial (Cook *et al*, 2005), a 22% lower risk of lung cancer was reported for the treatment group receiving 100 mg of aspirin every other day for 10 years, an amount similar to the 325 mg of aspirin once or twice a week in our NHS women. Fewer studies observed an increased risk of lung cancer for aspirin users. The only one with a significant result was a nested case-control study using the UK Health Improvement Network database in which a 53% increased risk of lung cancer was reported for men and women who received prescriptions for 150 mg or more per day of aspirin for at least 1 year (Hernandez-Diaz and Rodriguez, 2007). This increased risk is similar to what we observed for high quantity aspirin users in our cohort but the aspirin intake on which it was based is much lower.

## DISCUSSION

In this prospective study of women, we observed a 16% lower risk of lung cancer among regular users of one or two standard dose aspirin tablets per week and a 55% increased risk among those using 15 or more tablets per week for the past 2 years. It is difficult to interpret these diverse findings and to conclude whether one or both may be real effects or whether they are due to chance or residual confounding by smoking. The observed benefit from low quantity aspirin use may have been biased if women with heart disease were prescribed aspirin and also told to quit smoking. However, our results were unchanged when we excluded women who reported angina or a myocardial infarction. If aspirin is indeed beneficial in preventing lung cancer, it is anticipated to operate through anti-inflammatory pathways mediated by COX-2, the inducible form of the cyclooxygenase enzymes (Coussens and Werb, 2002). However, this requires higher doses of aspirin (Thun, 2000), as shown for colorectal cancer in several previous studies (Thun *et al*, 1993, Collet *et al*, 1999), including one within this NHS cohort (Chan *et al*, 2005). In this investigation, a lower risk of lung cancer was only observed for a quantity of aspirin use that was too low to affect COX-2. The lack of an increasing benefit with longer durations of aspirin use was also in contrast to the previous studies of colorectal cancer. If low-dose aspirin is indeed beneficial, it may act through pathways that do not involve inflammation and COX inhibition (Hanif *et al*, 1996).

No biologic mechanism is evident for our observed increased risk of lung cancer for women using 15 or more aspirin tablets per week, and it is likely at least partially the result of residual confounding, given the major impact of smoking on lung cancer and the positive association between smoking and aspirin use in this cohort. It is also possible that higher quantities of aspirin were used just prior to diagnosis to treat preclinical symptoms, though we did not find evidence to support this. Among the high quantity aspirin users, 12% of both the lung cancer cases and the non-cases had switched from no use or low quantity aspirin use in the previous assessment. The small size of the high quantity aspirin group prevented a more thorough examination of confounding by indication.

The prospective design of our study minimised the recall bias inherent in retrospective investigations, and the 24 years of biennial data collection provided updated information for accurate assessment of aspirin use, including status, quantity, frequency, and duration of use, and latency between aspirin exposure and lung cancer diagnosis. Self-reported aspirin use is prone to error, but is more likely to be accurate among the educated health professionals in this cohort. Moreover, such error would be random and would only have attenuated our results towards the null. The repeated biennial data collections also provided detailed and updated assessment of smoking, the primary confounder in lung cancer analyses. The importance of this confounding is demonstrated by the attenuation of the RR from 0.73 to 0.84 for women using 1–2 aspirin tablets per week when age at start of smoking, current cigarettes per day, and time since quit were added to the model. We acknowledge that measurement error remains in our smoking assessment, and it is possible that a more perfect assessment could further attenuate or nullify this association. On the other hand, we observed a lower risk of lung cancer for low quantity aspirin use even among the never smoking women in this cohort.

Our findings, together with those from previous clinical trials and prospective studies, do not offer consistent evidence that aspirin use is independently associated with risk of lung cancer. If there is indeed any effect of aspirin, future studies will require careful assessment of quantity and frequency of aspirin use, along with detailed histories of smoking and comorbidities.

## Figures and Tables

**Table 1:** Age-standardised characteristics^a^ of the study population of women by status and quantity of regular aspirin use over follow-up, 1980–2004

	**Aspirin use**						
				**Current tablets per week**			
	**Never**	**Past**	**Current**	**1–2**	**3–5**	**6–14**	**⩾15**
Age, years (mean)	53	60	57	56	57	59	55
Never smoker (%)	44	44	44	46	44	42	42
Past smoker (%)	35	40	38	38	38	39	35
Current smoker (%)	21	15	18	17	18	19	22
Cigarettes per day^b^ (mean)	19	18	19	18	18	19	20
Years since quit smoking^c^ (mean)	17	18	17	18	17	17	16
Pack-years of smoking^d^ (mean)	25	22	24	23	23	25	27
BMI, kg m^−2^ (mean)	25.3	26.2	25.8	25.6	25.6	26.3	26.6
Physical activity, hours per week (mean)	2.9	2.8	2.9	2.9	2.9	2.9	2.8
Post-menopausal (%)	69	69	69	68	69	70	70
oestrogen replacement therapy^e^ (%)	28	44	36	36	37	38	32

Abbreviation: BMI=body mass index.

a Calculated over all person–years of follow-up and standardised to the age distribution of the study population.

b Among current smokers.

c Among past smokers.

d Among past and current smokers.

e among post-menopausal women.

**Table 2:** Relative risks of lung cancer in women by status^a^ and quantity of aspirin use

			**Age-adjusted**	**Smoking-adjusted**
	**P-Y^b^**	**Cases**	**RR^c^ (95% CI^d^)**	**RR^e^ (95% CI^d^)**
Never aspirin use	418.0	236	1.00	1.00
Past aspirin use	501.9	405	0.84 (0.71–0.99)	0.97 (0.82–1.15)
Current aspirin use	1016.0	719	0.91 (0.78–1.06)	1.00 (0.86–1.16)
1–2 tablets per week	445.1	234	0.73 (0.60–0.87)	0.84 (0.70–1.02)
3–5 tablets per week	201.8	134	0.86 (0.70–1.07)	0.98 (0.79–1.21)
6–14 tablets per week	239.7	233	1.04 (0.86–1.25)	1.06 (0.88–1.28)
⩾15 tablets per week	65.6	65	1.58 (1.19–2.09)	1.55 (1.17–2.06)

Abbreviations: P-Y=person–years; RRs=relative risks; CI=confidence intervals.

a Current users reported a minimum of 1 tablet per week or 1 day per week of regular aspirin use over the previous 2 years; past users qualified as a current user sometime during cohort follow-up; never users never qualified as a current user.

b P-Y of follow-up, in thousands, from 1980–2004.

c RRs adjusted for age.

d 95% CI.

e RR adjusted for age, smoking status, age at start of smoking, years since quit smoking (past smokers), and cigarettes per day (current smokers).

**Table 3:** Relative risks of lung cancer in women using 1 to 2 or 15 or more aspirin tablets per week by duration of use and by latency period between aspirin assessment and lung cancer diagnosis

	**1–2 aspirin tablets per week**			**⩾15 aspirin tablets per week**		
*Duration of use* a	P-Y^b^	Cases	RR^c^ (95% CI^d^)	P-Y^b^	Cases	RR^c^ (95% CI^d^)
<2 years	202.5	119	0.86 (0.68–1.07)	27.0	33	1.68 (1.15–2.45)
2–5.9 years	153.8	81	0.87 (0.67–1.14)	21.1	20	1.44 (0.90–2.30)
⩾6 years	69.5	29	0.75 (0.50–1.19)	15.1	11	1.49 (0.81–2.76)
*P* for trend^e^			0.66			0.31
						
*Latency period* f	P-Y^g^	Cases	RR^c^ (95% CI^d^)	P-Y^g^	Cases	RR^c^ (95% CI^d^)
0 to <2 years^h^	445.1	234	0.84 (0.70–1.02)	65.6	65	1.55 (1.17–2.06)
2 to <4 years	397.7	238	0.88 (0.73–1.05)	57.8	53	1.24 (0.92–1.68)
4 to <6 years	359.5	240	0.91 (0.76–1.09)	50.3	51	1.19 (0.88–1.62)
6 to <8 years	323.2	256	1.02 (0.86–1.22)	43.2	45	1.15 (0.84–1.59)

Abbreviations: P-Y=person–years; RRs=relative risks; CI=confidence intervals.

a Duration was calculated as continuous years of aspirin use among current users.

b P-Y of follow-up, in thousands, from 1980 to 2004.

c RRs adjusted for age, smoking status, age at start of smoking (past and current smokers), years since quit smoking (past smokers), and cigarettes per day (current smokers).

d 95% CI.

e *P*-value for linear trend over median values within categories of years of aspirin use.

f During the latency periods for users of 1–2 aspirin tablets per week, women were censored if they reported ⩾15 tablets per week; during the latency periods for users of ⩾15 aspirin tablets per week, women were censored if they reported 1–2 tablets per week.

g P-Y of follow-up, in thousands, through 2004 and beginning in 1980 for a latency of 0 to <2 years; in 1982 for a latency of 2 to <4 years; in 1984 for a latency of 4 to <6 years; and in 1986 for a latency of 6 to <8 years.

h A latency of <2 years is the same as current users in Table 2 because aspirin use was assessed every 2 years.

**Table 4:** Descriptions and results from clinical trials and prospective studies of aspirin use and lung cancer

**Author, year trial or Cohort name**	**Design**	**Subjects**	**Cases**	**Lung cancer follow-up**	**Sex**	**Aspirin exposure or treatment**	**RR (95% CI)**
Peto *et al* (1988)	Clinical trial	5139	25	5–6 y treatment period	M	500 mg per d for 5–6 y	0.64 (0.30–1.41)
British Doctors Study							
Thun *et al* (1993)	Cohort	635 031	NA	6 y after assessment of	M	1–15 times per mo for 1 y	1.00 (0.88–1.13)
Cancer Prevention Study II				aspirin	F	16+ time per mo for 1 y	1.11 (0.98–1.25)
						1–15 times per mo for 1 y	0.73 (0.56–0.97)
						16+ times per mo for 1y	1.07 (0.88–1.30)
Lee *et al* (1994)	Clinical trial	22 071	128	5 y treatment period plus additional 5.9 y	M	325 mg per second d for 5 y	0.88 (0.62–1.25)
Physicians Health Study							
Langman *et al* (2000)	Nested cc	34 934	12 174	2 y after 1 y lag from aspirin assessment	M+F	2–5 prescriptions for 2 y	1.05 (0.89–1.24)
UK General Practice Research Database						7+ prescriptions for 2 y	0.84 (0.69–1.02)
Akhmedkhanov *et al* (2002)	Nested cc	808	81	12 y after 1 y lag from aspirin assessment	F	3+ times per wk for 6 mo	0.66 (0.34–1.28)
NYU Women's Health Study							
Holick *et al* (2003)	Cohort	49 383	328	10–14 y after assessment of aspirin	M	2+ times per wk for 2 y	1.13 (0.89–1.43)
Health Professionals Follow-up Study						2+ times per wk for 6 y	0.88 (0.58–1.34)
Cook *et al* (2005)	Clinical trial	39 876	205	10 y treatment period plus additional 1 y	F	100 mg per second d for 10 y	0.78 (0.59–1.03)
Women's Health Study							
Hayes *et al* (2006)	Cohort	27 162	403	10 y after assessment of aspirin	F	2–5 times per wk currently	0.85 (0.60–1.19)
Iowa Women's Health Study						6+ times per wk currently	1.08 (0.81–1.45)
Hernandez-Diaz and Rodriguez (2007)	Nested cc	10 000	4336	5.4 y after 1 y lag from aspirin assessment	M+F	1+ prescriptions for 1 y	1.15 (0.99–1.34)
UK Health Improvement Network						150+ mg per d for 1y	1.53 (1.22–1.92)
Feskanich *et al* (current study)	Cohort	109 348	1360	24 y during biennial reassessment of aspirin	F	1–2 tablets per wk for 2 y	0.84 (0.70–1.04)
Nurses' Health Study						15+ tablets per wk for 2 y	1.55 (1.17–2.06)

Abbreviations: cc=case control; d=day; wk=week; mo=month; y=year; M=male; F=female; RR=relative risk; CI=confidence interval; NA=not available.
